# Comparison of Deep SMAS Lift and MACS in Facelift: A Meta-Analysis

**DOI:** 10.3390/medicina62010112

**Published:** 2026-01-04

**Authors:** Alireza Pandkhahi, Najme Zare, Ali Mohammad Karimi, Alireza Ghafouriansamedanimashhad, Hamid Karimi, Andrea Alexander, Wolfram Trudo Knoefel, Sascha Vaghiri

**Affiliations:** 1 Medical Research School Duesseldorf, Heinrich-Heine-University Duesseldorf, Moorenstr. 5, 40225 Duesseldorf, Germany; alireza.kung1375@gmail.com; 2 Third Faculty of Medicine, Charles University, Ruská 87, 100 00 Prague, Czech Republic; najmezare2000@gmail.com (N.Z.); alireza.ghafourian@yahoo.com (A.G.); 3 First Faculty of Medicine, Charles University, Katerinská 32, 121 08 Prague, Czech Republic; 86551095@cuni.cz; 4 Plastic and Reconstructive Surgery, Faculty of Medicine, Iran University of Medical Sciences, Tehran 14166, Iran; 5 Department of Surgery (A), Heinrich-Heine-University, Medical Faculty and University Hospital Duesseldorf, Moorenstr. 5, 40225 Duesseldorf, Germany; andrea.alexander@med.uni-duesseldorf.de (A.A.); knoefel@med.uni-duesseldorf.de (W.T.K.); sascha.vaghiri@med.uni-duesseldorf.de (S.V.)

**Keywords:** face lift, rhytidectomy, SMAS, deep plane, surgical duration, complication

## Abstract

*Background and Objectives*: According to the PRISMA guidelines, this meta-analysis evaluates the outcomes of deep Superficial Musculoaponeurotic System (SMAS) lift versus Minimal Access Cranial Suspension (MACS) lift techniques in facelift surgery. *Materials and Methods*: We reviewed literature that included 20 studies involving a total of 7716 patients, of which four studies met our inclusion criteria, focusing on 286 patients (MACS n: 186 and SMAS n: 100). Our analysis assessed surgical duration, complications, pain levels, and aesthetic results. *Results*: Although both techniques yielded comparable aesthetic outcomes and postoperative complications, the MACS lift demonstrated a significantly shorter surgical duration (SMD = 2.05; 95% CI [1.61–2.48]; *p* < 0.00001, I^2^ = 0%) which has high impact on patient recovery, risk of complications and cost-effectiveness. *Conclusions*: The findings underscore the need for further research into varying techniques to solidify their efficacy and safety profiles.

## 1. Introduction

Face lift surgery (rhytidectomy) is a cornerstone of aesthetic plastic surgery and has evolved substantially over the past century. Early techniques focused primarily on skin excision and tightening; however, advances in anatomical understanding led to the development of deep-plane and SMAS-based approaches aimed at repositioning underlying soft tissues to achieve more durable and natural outcomes [1,2,3,4,5].

Contemporary facelift techniques therefore span a spectrum ranging from extended deep-plane/SMAS dissections to limited-access procedures designed to reduce scar length and operative morbidity while maintaining aesthetic efficacy [1,2,3,4,5,6].

Among commonly performed approaches, deep SMAS (deep-plane) techniques and the Minimal Access Cranial Suspension (MACS) lift represent two conceptually different strategies: the former relies on deeper dissection and tissue mobilization, whereas the latter uses limited access with vertical suspension sutures. Despite their widespread use, direct comparative evidence between these techniques remains limited, and available studies are heterogeneous in design, patient selection, outcome definitions, and reporting quality, which complicates evidence-based technique selection [3,4,5,7,8,9].

Accordingly, this systematic review and meta-analysis was conducted to compare deep SMAS (deep-plane) versus MACS lift techniques with special emphasis on operative time, postoperative complications, aesthetic results, and pain assessment. 

## 2. Materials and Methods

The presented meta-analysis was performed in strict adherence to the PRISMA (Preferred Reporting Items for Systematic Reviews and Meta-Analyses) Guidelines to ensure methodological rigor and reporting quality [10] (Table S1).

A comprehensive and systematic literature search was conducted to identify all published studies evaluating surgical outcomes of facelift procedures. The search strategy encompassed multiple electronic databases, including MEDLINE (PubMed), the Cochrane Central trials register, and Google scholar databases from their inception until October 2024 with no language or time restrictions. The following keywords “facelift”, “face lift”, “rhytidoplasty”, “rhytidectomy”, alongside modifiers like “primary”, “secondary”, “deep plane”, “deep SMAS”, and “MACS lift”, with the Boolean operators AND or OR were used to conduct the literature research.

Additional manual searches were performed through reference lists of relevant articles, key plastic surgery journals, to ensure maximal capture of eligible studies. No restrictions were initially placed on study design, publication year, or language during the primary search phase in order to minimize selection bias and ensure broad inclusion of the global facelift literature.

All studies retrieved during the initial search underwent an independent screening process performed by two reviewers (A.P., S.V.). Titles and abstracts were first evaluated to identify research focused on facelift, rhytidectomy, or facial rejuvenation surgery. Full-text assessment was subsequently conducted for all studies that met the preliminary eligibility criteria or when insufficient information was available from the abstract alone. Studies that did not involve surgical facelift procedures, those that reported only nonsurgical or adjunctive techniques (e.g., fillers, threads, or energy-based devices), review articles, expert opinions, and conference abstracts without extractable data were excluded at this stage.

For inclusion in the quantitative meta-analysis, studies were required to meet the following predefined criteria: (1) direct comparison of two distinct facelift techniques within the same study population (deep-plane vs. SMAS plication); (2) availability of extractable data related to postoperative outcomes such as complications, aesthetic results, patient satisfaction, or operative variables; and (3) a clearly described methodology that allowed assessment of surgical technique, patient selection, and outcome measurement. Randomized controlled trials, prospective cohort studies, retrospective comparative studies, and case–control designs were all eligible for inclusion, provided they met the above requirements. Studies reporting only a single surgical technique without a comparator group, or those lacking adequate detail on outcome measures, were excluded from the meta-analysis but documented in the narrative synthesis when relevant.

Any discrepancies between reviewers regarding study eligibility were resolved through discussion and consensus, with arbitration by a third reviewer (H.K.) when necessary. Data extraction was performed only after final agreement on inclusion. This rigorous selection process ensured that only high-quality comparative studies assessing two facelift techniques were incorporated into the quantitative analysis, thereby enhancing the reliability and scientific validity of the meta-analytic findings.

Studies that lacked comparative analyses (between the SMAS technique and the MACS lift) were excluded. We analyzed the cohort characteristics, number of patients, duration of surgery, complications (e.g., facial edema, hematoma, skin laxity, pain, nerve damage), and aesthetic results as reported by pain scores and patient satisfaction metrics. The primary outcome was the overall rate of complications. Secondary outcomes included OR time (min), the rate of postoperative hematoma, and the aesthetic-pain-related variables.

The overall quality and risk of bias assessment of the included non-randomized studies was performed using the ROBINS-I score as suggested in the Cochrane Handbook for Systematic Reviews of Interventions [11,12]. In summary, this well-established tool classifies non-randomized trials as low to high risk for bias using signal questions derived from seven potential different domains of bias at three time points (pre-intervention, at intervention, and post-intervention) in each study. Accordingly, two experienced authors (A.P., S.V.) independently evaluated each included study based on the proposed ROBINS-I domains. The certainty of evidence of significant outcomes was judged by the GRADE (The Grading of Recommendations, Assessment, Development, and Evaluation) criteria [13]. Again, discrepancies in risk of bias and certainty of evidence evaluation were resolved by consensus and if no agreement was reached, a senior author (H.K.) was asked for advice.

A pairwise meta-analysis of primary and secondary outcomes of interest was conducted using RevMan software (Version 5.3. Copenhagen: The Nordic Cochrane Centre, The Cochrane Collaboration, 2014). The odds ratio (OR) was calculated for dichotomous variables while standardized mean differences (SMD) were calculated for continuous parameters of interest. Summary estimates of treatment effect were calculated with 95% confidence interval (CI). The degree of heterogeneity among the included studies was measured by the I^2^ Index: 0–40% low heterogeneity, 30–60% moderate heterogeneity, 50–90% substantial heterogeneity, >75% considerable heterogeneity [12]. Summary estimates were calculated with a fixed-effects method in case of low or moderate heterogeneity (I^2^ < 60%). The significance threshold was determined by *p* < 0.05.

## 3. Results

Among the 20 identified papers, we selected four comparative articles meeting the requisite criteria, all published between 2005 and 2018, which included 286 patients (186 patients in the MACS lift group and 100 patients in the deep SMAS lift group) [14,15,16,17].

**Figure 1:**
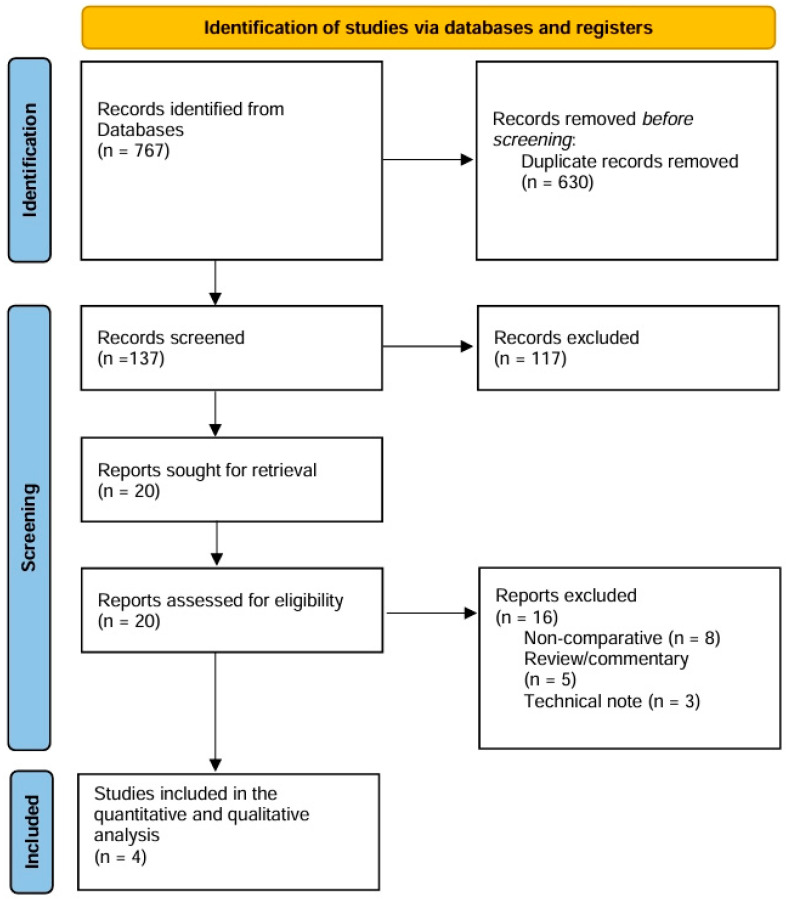
PRISMA flowchart of study identification and selection.

The PRISMA diagram of study identification and selection is demonstrated in Figure 1.

All analyzed studies were retrospective single-center studies, with varying follow-up periods ranging from 12 to 48 months. Typically, incisions were located around the ears, with some cases presenting anterior incisions. The patient demographics primarily featured females aged between 45 and 68 years (Table 1 and Table 2).

### 3.1. Technique Selection and Reported Indications

Across the four included retrospective single-center studies, explicit protocol-driven criteria for allocating patients to deep SMAS versus MACS were either not reported or not described in sufficient detail to allow extraction [14,15,16,17]. Zager et al. did not specify predefined anatomical or clinical indications guiding technique selection [14]. Prado et al. reported comparable baseline demographics between groups but did not report allocation criteria beyond surgeon choice [15]. Mast et al. included patients treated with both approaches, yet technique allocation and adjustment for baseline severity were not clearly described [16]. Buchanan et al. likewise did not report predefined indications; the deep SMAS group was older than the MACS group, suggesting potential selection based on perceived aging severity [17]. Overall, standardized indications for choosing one technique over the other could not be synthesized from the available evidence, and selection bias remains a key limitation when interpreting comparative outcomes [14,15,16,17].

### 3.2. Outcomes Analysis

#### 3.2.1. Primary Outcome

Overall morbidity

**Figure 2:**
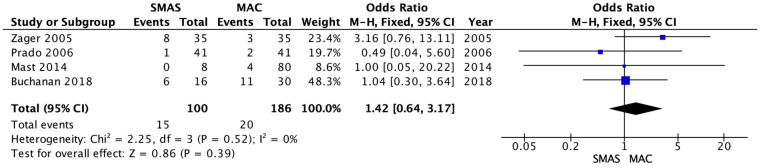
Forrest plot for primary outcome: overall morbidity [14,15,16,17].

The overall complications were mentioned in all four studies [14,15,16,17] with 286 patients as outlined in Figure 2. There were no significantly different records of overall postoperative complications in the SMAS and MACS lift groups. The occurrence of overall complications was 15 for the deep SMAS group and 20 for the MACS-lift group, respectively (OR = 1.42; 95% CI [0.64–3.17]; *p* = 0.39, I^2^ = 0%).

#### 3.2.2. Secondary Outcomes

Surgical Duration

**Figure 3:**
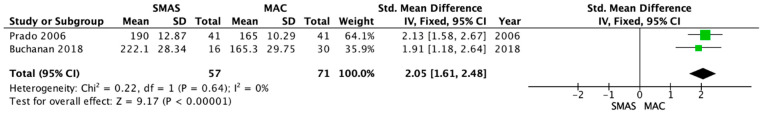
Forrest plot for secondary outcome: surgery duration [15,17].

The duration of surgery was provided in two studies [15,17] with a total of 128 patients. The MACS lift procedure resulted in a significantly shorter operation time when compared to the deep SMAS approach. Prado et al. [15] reported the mean ± SD of surgical duration for the SMAS group to be 190 ± 13 min and for the MACS-lift group to be 165 ± 10 min, respectively. Buchanan et al. [17] reported a surgery time of 222 ± 28 min for the SMAS group and 165 ± 30 min for the for MACS-lift group (SMD = 2.05; 95% CI [1.61–2.48]; *p* < 0.00001, I^2^ = 0%) (Figure 3). Based on GRADE, the certainty of evidence was moderate (Table S2).

### 3.3. Hematoma Formation

**Figure 4:**
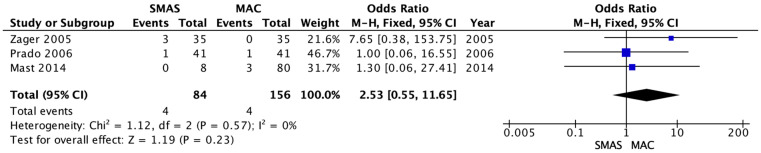
Forrest plot for secondary outcome: hematoma formation [14,15,16].

The occurrence of postoperative hematoma was reported in three studies [14,15,16] including 240 patients. The recorded incidence of postoperative hematoma was not significantly different between the deep SMAS and MACS lift cohorts. Zager et al. [14] reported hematoma formation in 3 SMAS group patients and zero in the MACS-lift group, respectively. Prado et al. [15] reported one hematoma occurrence in each group. Mast et al. [16] reported zero hematoma occurrence in the SMAS group and 3 in the MACS-lift group. (OR = 2.53; 95% CI [0.55–11.65]; *p* = 0.23, I^2^ = 0%) (Figure 4).

### 3.4. Non-Meta-Analyzed Parameters

Aesthetic outcomes: Aesthetic results were reported in two studies. Zager et al. [14] assessed outcomes using postoperative photographic evaluation with qualitative categorization by the operating surgeons (excellent, average, poor). Mast et al. [16] reported aesthetic outcomes based on patient satisfaction documented in medical records and categorized qualitatively (satisfied vs. disappointed). No standardized aesthetic scoring system was applied in either study. No statistically significant differences between the deep SMAS and MACS groups were reported in these assessments. Accordingly, the reported aesthetic outcomes reflect heterogeneous, non-validated assessment methods and should be interpreted descriptively rather than as directly comparable quantitative measures [14,16].

Pain assessment: Postoperative pain was evaluated in one study. Prado et al. [15] reported pain scores using a numerical rating scale; however, the scale range (e.g., 0–10) was not specified in the original publication. Mean pain scores were 4.1 ± 0.47 in the deep SMAS group and 6.1 ± 0.39 in the MACS group (*p* < 0.0001). As no scale definition was provided and no other included studies reported quantitative pain assessment, cross-study comparison and pooled analysis were not feasible [15].

Additional outcomes: Buchanan et al. [17] evaluated postoperative skin laxity and pleating qualitatively and reported no significant differences between techniques. Prado et al. [15] reported Strasser aesthetic scores at 1 and 24 months postoperatively, with no significant intergroup differences. One case of transient neuropraxia was reported in the MACS group by Mast et al. [16]; no permanent nerve injuries were documented across studies [14,15,16,17].

Edema/ecchymosis were reported in only one included study (Zager et al. [14]) and therefore could not be pooled quantitatively [14]. However, the results of this study demonstrated that the average amounts of ecchymosis and edema at 1 week and 2 weeks, respectively, were significantly reduced in favor of the minimal incision facelift group.

### 3.5. Risk of Bias Assessment

**Figure 5:**
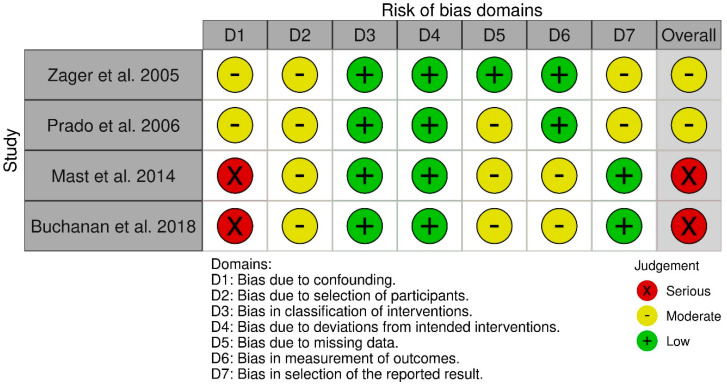
Risk of bias evaluation according to ROBINS-I [14,15,16,17].

The overall risk of bias was rated from moderate (Zager et al. [14] and Prado et al. [15]) to serious (Mast et al. [16] and Buchanan et al. [17]). The risk of bias due to confounding was judged as moderate in two studies [14,15] and serious in two studies [16,17]. All studies had a moderate risk of bias in the domain selection of participants and low risk of bias judgment in the classification of interventions and deviations from intended interventions domains, respectively [14,15,16,17]. Three studies demonstrated a moderate risk of bias due to missing data [15,16,17] and two studies [16,17] had moderate risk of bias in the domain measurement of outcomes. Bias in selection of the reported results was moderate in two studies [14,15]. The detailed risk of bias assessment is depicted in Figure 5.

## 4. Discussion

Facelift surgery has become an integral component of aesthetic medicine, addressing the physical manifestations of aging that are often a source of personal and social concern. Over the past century, the field has undergone substantial evolution, with numerous surgical techniques introduced and refined [1,2,3,4,5]. Facelift surgery, a practice that gained prominence over the last century, has continuously evolved as a critical intervention for combating the visible signs of aging [1,2,3,4,5,6]. The ideal face lift needs to have the longest efficacy, very few complications, and ultimately, the highest patient satisfaction [7,8,9]. Despite the proliferation of various surgical techniques, consensus regarding the most effective method remains elusive. This uncertainty stems primarily from the inherently subjective nature of aesthetic outcomes, which can differ significantly based on individual patient preferences and the nuanced artistry of the surgeon [5,8,9]. Moreover, variations in patient anatomy and the specific desires of individuals seeking surgical intervention add layers of complexity to the evaluation of facelift techniques. One other issue is the differences between men and women and their requirements [18]. Technical considerations in male facelift surgery differ from those in female patients and reported hematoma rates are higher among men [18]. The available literature reflects this variability, with a notable lack of standardized comparisons across different methodologies [19,20,21].

The findings of this review indicate that both deep SMAS and MACS lift techniques are viable options, with MACS associated with shorter operative time in the subset of studies reporting this outcome. However, the lack of high-quality studies limits definitive conclusions, and we suggest that we should focus on designing future studies with prospective, randomized designs, larger sample sizes, and standardized outcome measures. The influence of confounding variables (e.g., sex, blood pressure, surgical adjuncts) on secondary outcomes like hematoma and skin laxity warrants further investigation. This discussion explores the underlying principles of facelift surgery, compares leading surgical techniques, and highlights gaps in the literature that demand further investigation.

### 4.1. Comparative Studies and Gaps in the Literature

While numerous authors have documented their experience with various facelift techniques, studies that rigorously compare different approaches are surprisingly scarce. In our review of the literature during last 27 years, we identified 20 papers focusing on facelift methods, of which only four conducted comparative analyses of patients undergoing different surgical techniques [14,15,16,17]. Because these studies were performed as single-center studies with variability in methodology, our meta-analysis effectively consolidates the findings from these distinct approaches. With paucity of comparative studies, we recommend researchers and authors to conduct comparative prospective studies with controlled variables and proper follow-up periods to provide answers to these questions. It is obvious that the need for well-designed future studies to address these gaps, potentially supplemented by expert consensus methods, would have all related answers.

Our metanalysis showed that:

Incisional techniques frequently used in facelift surgeries tend to be periauricular, resulting in scars that, when healed, are often barely visible [1,3,9,22,23,24,25]. Our review confirms these findings, with many authors reporting favorable aesthetic outcomes. Whether the scars were concealed successfully is paramount, as it impacts patient satisfaction and the perceived quality of the surgical result.

### 4.2. Outcomes of Deep Plane SMAS vs. MACS Techniques

Our focused comparison of the Deep Plane SMAS (Superficial Musculoaponeurotic System) technique with the MACS (Minimal Access Cranial Suspension) lift revealed no significant differences in terms of aesthetic outcomes across various age groups. Kamer et al., Mohammadi et al. and Webster et al. have reported the same results [4,26,27]. However, none of the included retrospective studies provided a detailed or standardized description of the indications used to select one facelift technique over the other. As a result, potential selection bias related to surgeon preference, patient anatomy, or disease severity cannot be excluded.

In terms of postoperative complications, both techniques exhibited a comparable safety profile. However, data from Zager et al. [14] indicated a higher occurrence of edema and ecchymosis in the Deep SMAS group. Generally, the incidence of complications remained low, with only one reported case of neuropraxia out of 286 patients evaluated. Although Mast et al. [16] and Buchanan et al. [17] noted more complications associated with the MACS technique, the differences between the groups were minimal, non-significant and classified as mild and transient in nature.

### 4.3. The Aging Process

Facial aging is characterized by soft tissue atrophy, reduced skin elasticity, ligament weakening, and bone resorption, all of which contribute to sagging and volume loss. Decreased collagen and elastin, leading to thinner, less resilient skin that succumbs to gravitational forces. Effective facelift surgery must counteract these changes by repositioning tissues in a vertical and supero-lateral direction to restore youthful contours. Additionally, volume depletion should be addressed through fat transfer or filler injections, with fat transfer being the preferred method. Modern facelift techniques must adopt a holistic approach to achieve more natural and long-lasting rejuvenation.

### 4.4. Historical Background

The SubSMAS (Sub-Superficial Musculoaponeurotic System) dissection technique was first introduced by Tord Skoog in the 1970s as a significant advancement in facelift surgery. Before this innovation, traditional facelift techniques primarily focused on skin tightening, which often led to unnatural results and limited longevity. Skoog’s technique revolutionized facial rejuvenation by incorporating deeper tissue manipulation, specifically targeting the SMAS layer to achieve more natural, long-lasting outcomes.

His approach emphasized lifting and repositioning the deeper facial structures, rather than relying solely on skin traction. This not only improved the aesthetic durability of facelifts but also reduced complications such as unnatural skin tension and visible scarring. Skoog’s pioneering work laid the foundation for the modern deep-plane and extended SMAS facelift techniques, influencing the evolution of facial plastic and reconstructive surgery over the following decades.

Some authors believe that this technique may have more risk for nerve damage [19,20].

The MACS-lift (Minimal Access Cranial Suspension lift) was introduced by Dr. Patrick Tonnard and Dr. Alexis Verpaele in the early 2000s as a minimally invasive alternative to traditional facelift techniques. This method was developed to address facial aging with reduced scarring, shorter recovery time, and fewer complications compared to conventional facelifts.

Unlike traditional SMAS-based facelifts, the MACS-lift utilizes a short, preauricular incision (in front of the ear) and focuses on vertical vector suspension of facial tissues using purse-string sutures. These sutures tighten and elevate the underlying SMAS layer, leading to a natural-looking lift, particularly in the midface, jowls, and neck. The technique avoids extensive undermining and deep dissections, reducing risks of nerve damage and prolonged healing.

The SubSMAS technique typically involves a more extended incision, whereas the MACS lift uses a shorter incision.

The SubSMAS technique involves extensive dissection beneath the SMAS, whereas the MACS lift relies on limited undermining with a predominantly vertical lifting vector.

The SubSMAS technique allows lifting and repositioning of the SMAS and deeper tissue layers while MACS-lift does lift purse-string sutures for cranial suspension. SubSMAS method has longer recovery due to extensive dissection. The SubSMAS technique may be associated with a higher risk of nerve injury and prolonged postoperative swelling [19,20].

While both techniques aim to rejuvenate the face and restore a youthful contour, the SubSMAS dissection is often preferred for patients with significant skin laxity and deeper structural aging, whereas the MACS-lift is ideal for patients seeking a less invasive, quicker recovery option with moderate lifting effects [19,20].

In addition, recent anatomical studies, including the work of Minelli et al. [21], challenge the traditional concept of the Superficial Musculoaponeurotic System (SMAS) as a distinct and separate layer. Their research suggests that the SMAS is not an independent fascial structure but rather a continuum of fibrous and adipose tissue interwoven with facial muscles and retaining ligaments. Histological examinations indicate significant variability in SMAS composition across different facial regions. Some authors propose that what has been historically described as the SMAS is, in reality, a heterogeneous fibro-fatty network rather than a discrete anatomical entity. These findings have implications for facelift techniques, as they suggest that surgical approaches should focus more on individualized tissue composition and biomechanics rather than relying on a universally applied SMAS dissection strategy [21].

Expert commentary

In addition to the evidence synthesized in this review, surgical decision-making in facelift procedures is influenced by factors such as surgeon experience, patient anatomy, and individual preferences regarding recovery time and scar placement [28]. These considerations reflect general clinical practice and are not derived from the meta-analytic data presented in this study. Accordingly, they should be interpreted as contextual clinical perspective rather than evidence-based findings of the systematic review.

In clinical practice, technique selection is often influenced by patient-specific anatomical factors, such as the degree of midface descent, soft tissue thickness, and skin laxity. These considerations are discussed in the broader literature but were not systematically evaluated in the studies included in this review and therefore should not be interpreted as evidence-based findings of the present analysis [29].

Surgical procedures that involve extensive incisions and wide tissue undermining—such as rhytidectomy—tend to reveal more pronounced differences in healing characteristics between sexes and across ethnic groups compared with limited-dissection procedures [30]. In particular, Asian male patients often present unique challenges owing to their comparatively thicker, denser skin and a greater tendency toward hypertrophic or conspicuous scar formation. Furthermore, Asians generally exhibit a more developed subdermal vascular plexus, with numerous vertically oriented perforators. During flap elevation, these vessels are more prone to bleeding than in Caucasian patients, which may prolong operative time and increase the risk of postoperative hematoma. These observations are consistent with literature demonstrating increased fibroproliferative scar responses and prolonged hyperemia during scar maturation in Asian skin compared to Caucasian skin [30,31]. 

Aesthetic preferences also differ substantially between ethnic populations due to variations in craniofacial skeletal morphology. Caucasian patients frequently seek outcomes that enhance facial projection and highlight three-dimensional contours, including prominence of the malar region. Conversely, many Asian patients prefer a smoother, less angular facial contour and generally do not desire additional accentuation of the zygomatic arch or malar eminence, which are often already relatively prominent. These differences reflect documented distinctions in objective facial-shape criteria and subjective aesthetic ideals between Caucasian and East Asian populations [31,32].

These anatomical and cultural considerations necessitate tailored surgical planning, not only with respect to age-related tissue characteristics but also accounting for sex-specific factors and ethnic variations in skin biology, vascular anatomy, healing patterns, and aesthetic ideals. Such individualized approaches are essential for optimizing both surgical safety and patient satisfaction across diverse populations.

### 4.5. Limitations and Future Directions

Despite the insights garnered from our study, some limitations cannot be overlooked. The relatively small number of retrospective and comparative studies (during last 27 years) presents challenges in establishing broader generalizability of the results. Nevertheless, the aggregate patient cohort across the selected studies was sufficiently robust, yielding low heterogeneity (I^2^ = 0%) of pooled results, which reinforces the reliability of our conclusions.

The limitations inherent in our meta-analysis underscore the necessity for further, larger-scale studies to substantiate the findings within the context of evolving facelift techniques. Within the limitations of retrospective comparative data, both deep SMAS and MACS lift techniques demonstrated comparable short-term safety profiles. The influence of confounding variables (e.g., sex, blood pressure, surgical adjuncts) on secondary outcomes like hematoma and skin laxity warrants further investigation. Future research should also consider a broader range of aesthetic and complication data, as well as patient-centered outcomes such as quality of life and satisfaction levels post-surgery.

## 5. Conclusions

In summary, our meta-analysis provides compelling evidence that the MACS lift technique offers comparable aesthetic results to that of the Deep Plane SMAS method, coupled with the added benefits of shortened surgical times and similar complication rates. While the available literature presents some limitations, our findings lay the groundwork for future studies aimed at refining surgical practices in facial rejuvenation and addressing the complex needs of the aging population. As the field progresses, it is imperative that both surgeons and researchers continuously evaluate the efficacy of facelift techniques in tandem with emerging aesthetic ideals and technological advancements, ensuring that patient outcomes remain at the forefront of surgical evolution.

## Figures and Tables

**Table 1:** Study characteristics.

Author	Year	Origin	Study Period	Study Design	Total Sample Size	Follow-Up Period (Months)
Zager et al. [14]	2005	United States	NA	Single-center, retrospective	70	12
Prado et al. [15]	2006	Chile	July 2002–January 2005	Single-center, retrospective	82	24
Mast et al. [16]	2014	United States	August 2006–October 2011	Single-center, retrospective	88	8.5 ± 9.1
Buchanan et al. [17]	2018	United States	January 2013–December 2015	Single-center, retrospective	46	6.44 ± 3.6

NA: not available.

**Table 2:** Patient group characteristics.

Author	Group(s)	No. of Patients	Age (Years) Mean ± SD	Gender M/F	BMI (Kg/m^2^) Mean ± SD
Zager et al. [14]	SMAS	35	56.3 ± 5.24	NA	NA
	MACS	35	54.4 ± 6.15	NA	NA
Prado et al. [15]	SMAS	41	47 ± 2.92	M: 1 F: 40	NA
	MACS	41	47 ± 2.92	M: 1 F: 40	NA
Mast et al. [16]	SMAS	8	59	M: 3 F: 85	25
	MACS	80			
Buchanan et al. [17]	SMAS	16	64.8 ± 4.69	M: 1 F: 15	24.2 ± 2.12
	MACS	30	60.2 ± 5.83	M: 2 F: 28	23.5 ± 3.01

BMI: body mass index, MACS: minimal access cranial suspension, NA: not available, SMAS: superficial muscular aponeurotic system.

## Data Availability

No new data were created or analyzed in this study.

## Supplementary Materials

The following supporting information can be downloaded at: https://www.mdpi.com/article/10.3390/medicina62010112/s1, Table S1: Checklist [10]; Table S2: The GRADE Certainty assessment for the significant outcomes [13].
