# Supplementary material for: Comparison of Deep SMAS Lift and MACS in Facelift: A Meta-Analysis
Source: Medicina (Kaunas). 2026 Jan 4;62(1):112. doi: 10.3390/medicina62010112 (PMC12843705; doi:10.3390/medicina62010112)
Supplement: Supplementary file 1 [file medicina-62-00112-s001.zip › medicina-3997128-table S2.pdf]

Table S2 The GRADE Certainty assessment for the significant outcomes

Outcome	No. of studies	**No. of included patients**		SMD [95 % CI]	**Quality assessment**					Quality
		SMAS	MACS		Risk of bias[a]	Inconsistency	Indirectness	Imprecision	Publication bias	
Surgery duration (min)	2 [11,13]	57	71	2.05 [1.61-2.48]	Serious	Not serious	No indirectness	No imprecision	NA	Moderate

MACS: minimal access cranial suspension, NA: not applicable, SMD: standardized mean difference, SMAS: superficial muscular aponeurotic system

[a] Risk of bias assessed using the ROBINS-I tool
